# Small size gold nanoparticles enhance apoptosis-induced by cold atmospheric plasma via depletion of intracellular GSH and modification of oxidative stress

**DOI:** 10.1038/s41420-020-00314-x

**Published:** 2020-09-10

**Authors:** Paras Jawaid, Mati Ur Rehman, Qing-Li Zhao, Masaki Misawa, Kenji Ishikawa, Masaru Hori, Tadamichi Shimizu, Jun-ichi Saitoh, Kyo Noguchi, Takashi Kondo

**Affiliations:** 1 Department of Radiology, Graduate School of Medicine and Pharmaceutical Sciences University of Toyama, Toyama, Japan; 2 Theranostic Devices Research Group, Health Research Institute, National Institute of Advanced Industrial Science and Technology (AIST), Takamatsu, Japan; 3 Center for Low-temperature Plasma Science, Nagoya University, Nagoya, Japan; 4 Department of Dermatology, Graduate School of Medicine and Pharmaceutical Sciences University of Toyama, Toyama, Japan

**Keywords:** Radiotherapy, Apoptosis

## Abstract

Gold nanoparticles (Au-NPs) have attracted attention as a promising sensitizer owing to their high atomic number (Z), and because they are considered fully multifunctional, they are preferred over other metal nanoparticles. Cold atmospheric plasma (CAP) has also recently gained attention, especially for cancer treatment, by inducing apoptosis through the formation of reactive oxygen species (ROS). In this study, the activity of different sized Au-NPs with helium-based CAP (He-CAP) was analyzed, and the underlying mechanism was investigated. Treating cells with only small Au-NPs (2 nm) significantly enhanced He-CAP-induced apoptosis. In comparison, 40 nm and 100 nm Au-NPs failed to enhance cell death. Mechanistically, the synergistic enhancement was due to 2 nm Au-NPs-induced decrease in intracellular glutathione, which led to the generation of intracellular ROS. He-CAP markedly induced ROS generation in an aqueous medium; however, treatment with He-CAP alone did not induce intracellular ROS formation. In contrast, the combined treatment significantly enhanced the intracellular formation of superoxide (O_2_^• −^) and hydroxyl radical (^•^OH). These findings indicate the potential therapeutic use of Au-NPs in combination with CAP and further clarify the role of Au-NPs in He-CAP-aided therapies.

## Introduction

Nanotechnology has gained tremendous attention because of its application in cancer therapy^1^. Nanoparticles consisting of high atomic (Z) number elements (e.g., Gold nanoparticles (Au-NPs) are being intensely investigated for use in the treatment of cancer. Among the many nanomaterials tested, Au-NPs have gained popularity for several reasons: they are considered to be fully multifunctional, enabling the combination of various activities in a single molecular package^2^. In medicine, Au-NPs are widely applied to intensify the therapeutic efficacy of drugs by modifying their uptake into the cells. Au-NPs of sizes 10–30 nm tend to localized in cancer cells more rapidly than the Au-NPs of larger sizes because of enhanced permeability and retention (EPR) effect^3,4^.

Plasma medicine has gained increasing attention based on its application in interdisciplinary fields^5^. In plasma medicine, “plasma oncology,” i.e., the use of cold atmospheric plasma (CAP), ionized gas at near room temperature, has shown potential for the treatment of cancer. CAP can generate many biologically active agents, including radicals, positive and negative ions, and several other elements^6–8^, in either the liquid phase or inside of cells^5^. Anticancer effects of CAP are mainly executed via the formation of reactive oxygen species (ROS) and/or reactive nitrogen species (RNS)^8^. Although cancer cells are more susceptible to ROS-associated stimuli, the clinical application of CAP remains challenging because of biological and technical problems. Recently, synergistic anticancer effects of Au-NPs and CAP have emerged as a promising combined treatment approach, compared with either treatment alone, with the capability of high nanoparticle uptake, because membrane permeability makes cancer cells more susceptible than healthy cells^9^. Several groups have reported the efficacy of Au-NPs with CAP in breast cancer, colorectal cancer, and glioblastoma^10–12^. However, these studies did not address the underlying mechanism involved in the enhancement of cell death. The effects of metal nanoparticles can vary depending on their size and physical properties. It is essential to address that the effects of Au-NPs can vary depending on various factors. In a previous study, small-sized platinum nanoparticles (Pt-NPs) quenched helium (He)-based CAP (He-CAP) induced ROS, which inhibited the ROS-mediated pathway of apoptosis that had been induced by He-CAP^13^.

Therefore, the present study was executed to determine the effects of various sizes of Au-NPs on He-CAP-induced apoptosis and its underlying molecular mechanism. In addition, changes in He-CAP effects after Au-NPs-induced ROS production and DNA damage were also determined.

## Materials and methods

### Preparation of Au-NPs

Au-NPs of sizes 2 nm, 40 nm, and 100 nm were obtained from BBI. Solutions (British Biocell International Limited, United Kingdom). Au-NPs were suspended in H_2_O and no preservative, residual chemical left from the manufacturer. Au-NPs were diluted 1:5 in the Roswell Park Memorial Institute (RPMI) 1640 cell culture medium. The final gold concentration for 2 nm is 2.5 μg/ml. For comparison, 40 and 100 nm Au-NPs were used at a final gold concentration of 11 μg/ml.

### Transmission electron microscopy

An aliquot of 1 µL solution of each sample was diluted with water by 1/10, and was placed on a collodion membrane transmission electron microscopy (TEM) grid (COL-C15, Ohken Shoji, Japan). After the liquid was dried away in a vacuum chamber, 40 nm and 100 nm samples were observed by a TEM microscope (S4800, Hitachi) with a magnification 450,000–500,000 at 10 kV acceleration voltage, without platinum plating. Sample of 2 nm was observed by a TEM microscope (JREM-ARM200F-G, JEOL Ltd.) at 200 kV electron acceleration voltage.

### Cell culture

Human myelomonocytic lymphoma U937 cells were obtained from the Human Sciences Research Resource Bank (Japan Human Sciences Foundation, Tokyo, Japan). The U937 cells were grown in RPMI 1640 culture medium with 10% heat-inactivated fetal bovine serum at 37 °C in humidified air with 5% CO_2_.

### Cold atmospheric helium-based plasma treatment

U937 cells (0.5 × 10^6^) were cultured in a 24 well plate with 1 ml of RPMI 1640 with or without Au-NPs Cells were irradiated at a distance of 1 cm from the tip of the plasma jet tube to the solution surface. He-CAP was carried out at room temperature by a CAP system (PN-120TPG, NU Global, Japan), which has a gas flow controller, a voltage power supply, and a handpiece of the plasma jet. The diameter of the dielectric tube was 1 and 2 mm, respectively. The dielectric tube constructed with an inner micro hollow type electrode and an outer dielectric barrier electrode. The plasma jet was ~20 mm in length. For the generation of Plasma with He gas at a flow rate of 2 L/min, and the gas temperature below 350 K was applied in this study^13^. Cells were harvested at the indicated time periods after treatment.

### Cell viability

Cell counting kit (CCK-8) from (Dojindo Laboratories Co., Ltd., Kumamoto, Japan) was used to perform cell counting assay. After 24 h of combined treatment 100 μl of RPMI containing cells was added into 96-well plate with 10 μl CCK-8 per well; incubate for 2 h at 37 °C in 5% CO_2_ then the absorbance was observed at 450 nm by using Microplate Reader (Bio-Rad Laboratories, Inc. Hercules, CA, USA).

### DNA fragmentation assay

For the detection of apoptosis, the percentage of the DNA fragmentation was assessed 24 h post treatment using the method of Sellins and Cohen with minor modification^14^. Approximately 2 × 10^6^ cells were lysed, using 200 μl of lysis buffer, incubate for 20 min on ice, then centrifuged at 13,000 × *g* for 10 min. The DNA sample is in the supernatant, and the pellet were precipitated in 25% trichloroacetic acid (TCA) at 4 °C overnight. Centrifuge the samples then discard the supernatant and hydrolysis in 5% TCA at 90 °C for 20 min then quantified it in the diphenylamine reagent overnight at 37 °C. The percentage of fragmented DNA in each sample was calculated as the amount of DNA in the supernatant divided by total DNA for that sample (supernatant plus pellet)^13^.

### Detection of apoptosis using Annexin V-FITC/ PI staining

The early apoptosis and secondary necrosis were determined after the 24 h of treatment. Cells were collected and washed with cold PBS at 4 °C then centrifuged at 1200 rpm for 3 min; the resulting pellet then mixed with the binding buffer Propidium iodide (PI; 5 μl) and fluorescein isothiocyanate (FITC)-labeled annexin V (5 μl), (Immunotech, Marseille, France) were added to the suspension and mixed gently, then incubate for 20 min at 4 °C in the dark. Cells were analyzed by using Flow cytometry (Epics XL, Beckman-Coulter, Miami, FL).

### Morphological detection of apoptosis by Giemsa staining

The morphological changes in the treated and control cells examined by Giemsa staining. After 24 h of incubation at 37 °C, the cells were collected and washed with PBS, then fixed with methanol and acetic acid (3:1) and spread on the glass slides. Staining was done by using 5% Giemsa solution (pH 6.8) for 15 min.

### Cell cycle analysis

After 24 h of treatment administration, cells were fixed with pre-chilled 70% ethanol and left overnight at −20 °C. Subsequently, fixed cells were treated with 0.25 mg/ml RNase A (Nacalai Tesque, Kyoto, Japan) and 50 μg/ml PI in PBS following adequate washing with PBS. The samples run by using a flow cytometer.

### Assessment of intracellular ROS generation

Intracellular ROS generation was evaluated using dichlorofluorescein diacetate (DCFH-DA; Molecular Probes, Eugene, OR) and superoxide (O_2_^• −^) generation sensitive dye, HE (Molecular Probes, Eugene, OR) and 2-[6-(4^/^-hydroxy)phenoxy-3*H*-xanthan-3-on-9-yl] benzoic (HPF) fluorescence probes were used to determine some species of intracellular ROS (ONOO^−^, ^•^OH) generation in terms of an increase fluorescence and that are highly resistant to autoxidation. The cells were treated with or without Au-NPs then immediately exposed to He-CAP for 2 min, DCFH-DA was added at a final concentration of 10 μM, HE and HPF were added at a final concentration of 5 μM after 3 h of post treatment. The fraction of fluorescence positive cells was measured by flow cytometry as the proportion of cells containing intracellular ROS.

### Assessment of intracellular GSH

Glutathione (GSH) kit (Abcam; ab112132) was used to measure intracellular GSH. Cells were exposed to He-CAP with or without the presence of Au-NPs. After 18 h of incubation, the cells were collected and washed with PBS, then loaded with green dye (50 nmol/l) for 30 min. The fluorescence intensity of green dye was detected with a flow cytometer at FL1 channel.

### Assessment of mitochondrial transmembrane potential

The tetramethylrhodamine methyl ester (TMRM) accumulated electrophoretically in mitochondria in response to mitochondrial membrane potential (MMP), which then released upon loss of MMP. After Au-NPs and He-CAP treatments, the treated cells were incubated at 37 °C for 18 h, collected, washed with PBS, and centrifuged at 1200 rpm for 3 min. Then the cells were stained with the 10 nM cationic fluorophore, TMRM from (Molecular Probes, Eugene, OR), for 15 min at 37 °C in 1 ml of PBS, followed by the immediate flow cytometry of red TMRM fluorescence (excitation at 488 nm; emission at 575 nm).

### Determination of intracellular Ca^2+^ ions

To monitor the effect of He-CAP on intracellular Ca^2^ homeostasis, intracellular free Ca^2+^ was measured using the calcium probe Fluo-3/AM (Dojindo Laboratories Co., Ltd., Kumamoto, Japan). Cells were harvested 18 h after treatment and then incubated with 5 μM Fluo-3/AM for 30 min at 37 °C. Wash the cells with PBS to remove excess Fluo-3/AM. The intensity of Ca^2+^ was measured by flow cytometry.

### Detection of Fas on the cell surface

After 18 h of treatment, cells were washed twice with cold PBS, re-suspended in 20 μl of washing buffer containing 2.5 μg/ml of FITC-conjugated anti-Fas monoclonal antibody (clone: UB3, MBL, Nagoya, Japan) and incubated for 30 min at room temperature. Flow cytometry was used to analyze the data.

### Measurement of caspase-8 activity

A FLICE/caspase-8 colorimetric protease assay kit (MBL, Nagoya, Japan) was used according to the manufacturer’s instructions. After 18 h, the cells were harvested, lysed, and the protein lysate was collected. The 200 μg protein samples mixed with 50 μl of 10 mM dithiothreitol at a final concentration of 200 μM. Incubate the mixtures at 37 °C for 2 h; then, the activity was measured at 405 nm using a spectrophotometer (Beckman Instruments Inc., Fullerton, CA).

### Western blot

At the indicated time period, collect and washed the cells with cold PBS. Cells were lysed at a density of 2.5 × 10^6^ cells/70 μl of RIPA buffer (50 mM Tris-HCl, 150 mM NaCl, 1% Nonidet P-40 (v/v), 1% sodium deoxycholate, 0.05% SDS, 1 μg of each aprotinin, pepstatin, and leupeptin and 1 mM phenylmethylsulfonyl fluoride) for 20 min. The lysates were centrifuged at 12,000 × *g* for 10 min at 4 °C after sonification, and the protein content in the supernatant was measured using the Bio-Rad protein assay kit (Bio-Rad, Hercules, CA). Protein lysates were denatured at 96 °C for 5 min after mixing with SDS-loading buffers, applied on an SDS-polyacrylamide gel for electrophoresis, and transferred to nitrocellulose membrane. Western blot analysis was performed to detect expression using specific antibodies against H2AX (#9718), Caspase-3 (#9662), XIAP (#2042), Bid (#2002), Bax (#2772), Bcl-xL (#2762), Fas (#4233), Cleaved Caspase-8 (#9496), JNK (#9252), Phospo JNK (#9255), Erk (#5013), Phospho Erk (#4377), Phospo-p38 (#9211) were from Cell Signalling Technology Inc. β-actin (AC-15, A5441) was obtained from Sigma Aldrich Inc. All primary antibodies were used 1:1000 dilution for experiment except β-actin. β-actin was used 1:10,000 dilution. Blots were probed with either secondary horseradish peroxide-conjugated anti-rabbit (#7074) or anti-mouse IgG (#7076) antibodies (1:1000 dilution) obtained from Cell Signalling Technology Inc. Band signals were visualized on a LI-COR image analyzer (Linclon, Nebraska, USA) by using either chemi-Lumi One L (Nacalai Tesque, Kyoto, Japan) or ImmunoStar LD (Wako, Japan) detection reagents.

### Statistical analysis

The data are shown as the mean ± standard error mean. For all assay conditions, three independents experiments were conducted. Data were analyzed with Student’s *t* test. For multiple group analysis and interaction, one-way analysis of variance(ANOVA) and two-way ANOVA, followed by Bonferroni’s multiple comparison test were used. Differences were statistically significant at *P* value <0.05. Both statistical tests and *P* values are shown in either figure or figure legends.

## Results

### Size-dependent effect of Au-NPs on He-CAP-induced apoptosis

**Fig. 1:**
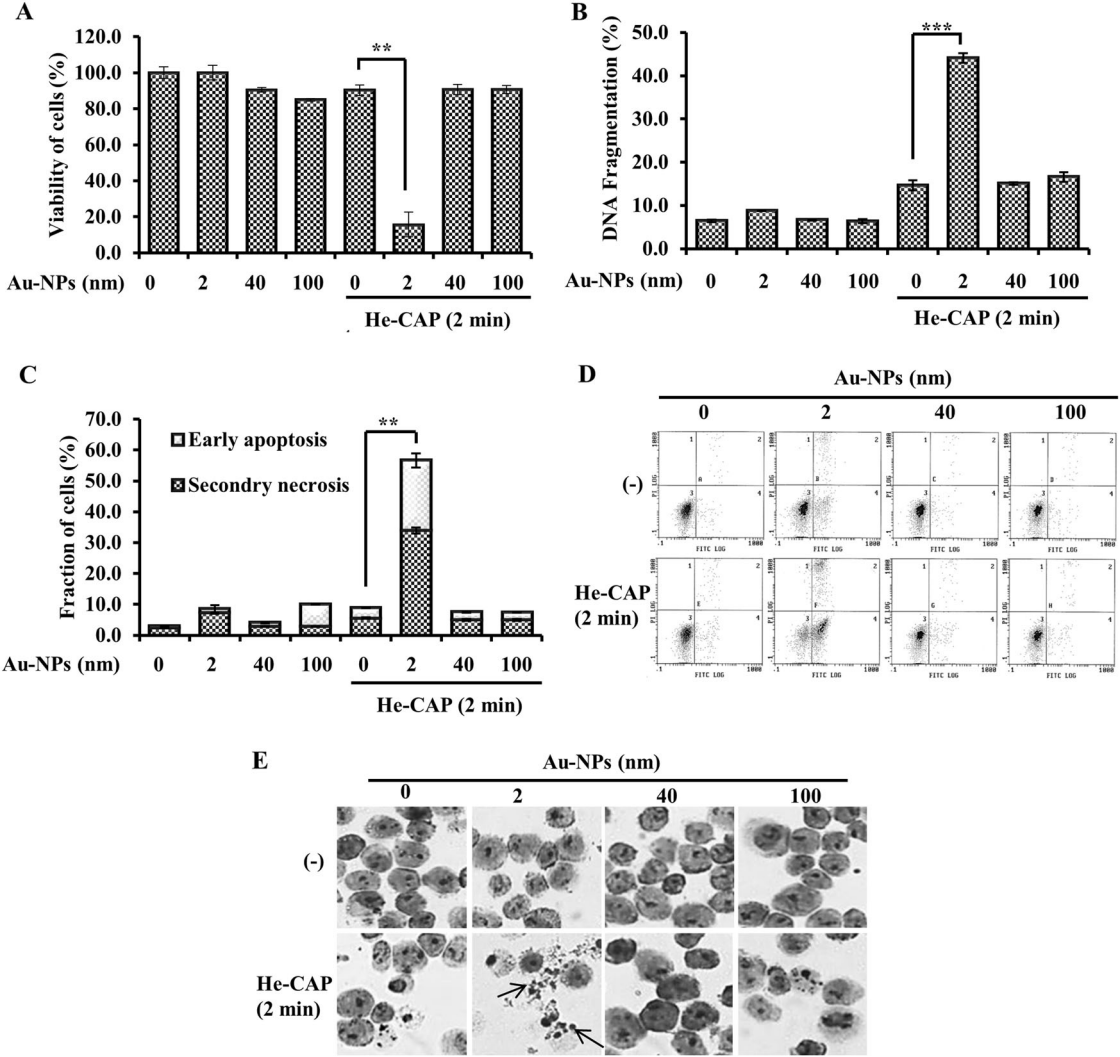
Effects of different sizes of Au-NPs, i.e., 2 nm, 40 nm, and 100 nm on He-CAP-induced cell death. **a** Cells were treated with He-CAP for 2 min in the presence or absence of Au-NPs, and cell viability was analyzed after 24 h. Results are represented as means ± S.E.M. of three independent experiments, **P < 0.001 vs the He-CAP alone group evaluated by Student’s *t* test (S.E.M. is indicated by bars). **b** DNA fragmentation assay was carried out 24 h after He-CAP exposure. Results are represented as means ± S.E.M. of three independent experiments, ***P < 0.0001 vs the He-CAP alone group evaluated by Student’s *t* test (S.E.M. is indicated by bars). **c** Effects of the Au-NPs on He-CAP-induced early apoptosis and secondary necrosis. Cells were treated with He-CAP with or without Au-NPs. The percentages of cells in early apoptosis and secondary necrosis were analyzed by flow cytometry 24 h after He-CAP treatment. Results are represented as means ± S.E.M. of three independent experiments, **P < 0.001 vs the He-CAP alone group evaluated by Student’s *t* test (S.E.M. is indicated by bars). **d** Representative flow cytometry histogram based on annexin V-FITC and PI-stained cells. **e** Assessment of the morphological changes during He-CAP-induced apoptosis. The sign of apoptosis was dyed by Giemsa stain, and then the cells were examined under a microscope at 400x magnification. One representative photomicrograph is shown here, the arrowhead shows apoptotic cells.

Au-NPs of sizes 2, 40, and 100 nm from BBI. solution were imaged by TEM to determine size and morphology compared with the manufacturer’s specification (Supplementary Fig. 1). The size-dependent ability of Au-NPs to synergistically enhance cell death induced by He-CAP was evaluated in U937 cells. He-CAP exposure for 2 min in the presence of small Au-NPs (2 nm) incubated for 24 h significantly suppressed the viability of the cells (Fig. 1a). Similarly, the DNA fragmentation was slightly increased in the cells He-CAP treatment alone, reaching up to 40.0 ± 5.0% in the presence of the Au-NPs (2 nm) (Fig. 1b). Then, membrane changes indicative of apoptosis or necrosis of the U937 cells were evaluated by determining the uptake of annexin V-FITC/PI double staining. Small Au-NPs (2 nm) and He-CAP caused a more significant increase in early apoptosis and secondary necrosis than He-CAP alone or larger Au-NPs (40 and 100 nm) (Fig. 1c, d). The combined treatment exhibited strong synergic apoptosis induction after incubation with cells for 24 h. Moreover, 2 nm Au-NPs/He-CAP treatments caused markedly enhanced typical morphological changes of apoptosis, such as nuclear condensation and fragmentation and cytoplasmic aggregation, compared with either treatment alone or others combined. No sign of necrosis or other types of cell death was observed (Fig. 1e). As Au-NPs (2 nm) showed synergetic enhancement of apoptosis, they were used in subsequent experiments.

### Effects of Au-NPs on He-CAP-induced cell cycle distribution

**Fig. 2:**
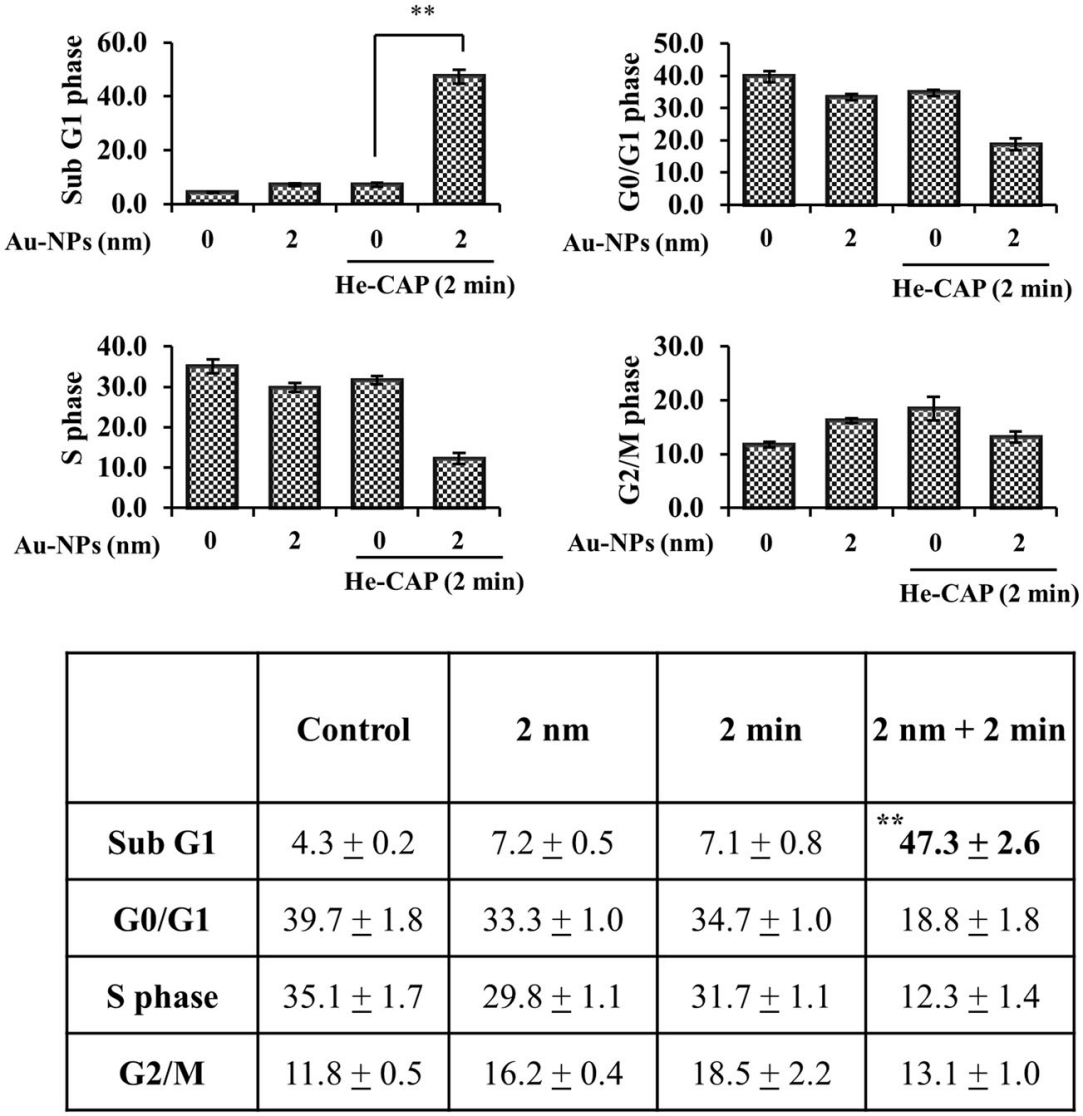
Cell cycle analysis by flow cytometry. Results are represented as means ± S.E.M. of three independent experiments, **P < 0.001 vs the He-CAP alone group evaluated by Student’s *t* test (S.E.M. is indicated by bars).

The synergistic group induced cycle distributions are shown in (Fig. 2). The He-CAP alone and Au-NPs alone groups showed no marked increase in the sub-G1 fraction compared with that of the control group. In contrast, the combined treatment caused a significant increase in the sub-G1 fraction compared with that of both the control and alone groups. Although Au-NPs alone and He-CAP treatment alone causes a slight increase in G2/M levels, however, this increase is not significant compared to the control group. In the combined treatment with Au-NPs and He-CAP G2/M levels were reduced to 13.1 ± 1.0 from 16.2 ± 0.4 and 18.5 ± 2.2, respectively. The increase in the sub-G1 fraction following combined treatment was significantly increased than either of treatment alone with a decrease in G1 and G2/M phases levels, which is due to the induction of apoptosis.

### Upregulation of ROS formation and DNA damage in response to GSH depletion caused by Au-NPs

**Fig. 3:**
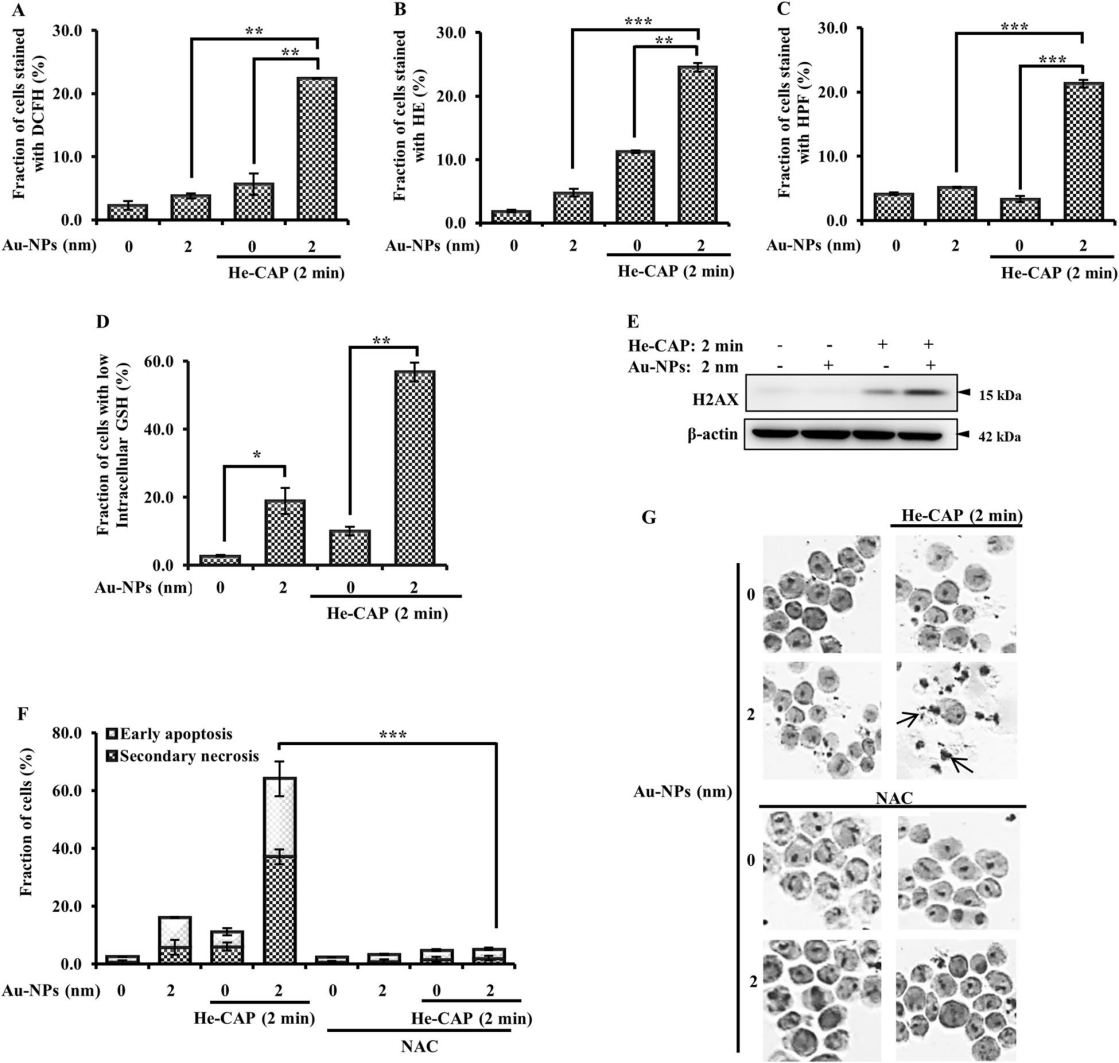
Effects of Au-NPs on He-CAP induced intracellular ROS production. The percentages of cells with elevated species of ROS were analyzed 3 h after He-CAP by flow cytometry. **a** DCFH-DA staining, **b** HE staining, **c** HPF staining. Results are represented as means ± S.E.M. of three independent experiments, **P < 0.001 and ***P < 0.0001 vs either treatment alone as determined by one-way ANOVA with Bonferroni’s multiple comparison test (S.E.M. is indicated by bars). **d** Intracellular GSH level, as measured using a G.S.H. kit and analyzed by flow cytometry. Results are represented as means ± S.E.M. of three independent experiments, *P < 0.05 compared with the control group and, **P < 0.001 compared to the He-CAP alone group evaluated by Student’s *t* test (S.E.M. is indicated by bars). **e** Assessment of DNA damage. The extent of H2AX phosphorylation was determined immediately after He-CAP treatment in the presence or absence of Au-NPs. β-actin was used to normalize the expression level of each sample. Blots were cropped, full-length blots are presented in Supplementary Fig. 2. **f** Assessment of apoptotic cell death in the presence of NAC, Annexin V-FITC/PI. Results are represented as means ± S.E.M. of three independent experiments, ***P < 0.0001 vs the combined treatment group as determined by two-way ANOVA with Bonferroni posttest (S.E.M. is indicated by bars). **g** Giemsa staining under a microscope at ×400 magnification. One representative photomicrograph is shown here, with the arrowhead showing apoptotic cells.

ROS generation is the most notable factor in CAP-treated cancer cells. Different species of ROS were detected using specific probes, including DCFH-DA, hydroethidine (HE), and HPF. Au-NPs individual treatment did not show a marked increase in ROS formation. He-CAP treatment alone slightly increased the intensity of DCFH-DA and HE. However, the flow cytometry results showed significantly enhanced ROS generation 3 h after combined treatment administration (Fig. 3a–c). This finding shows that the intracellular ROS level was markedly increased in the synergistic group and enhanced the overall biological effects of He-CAP. Subsequently, intracellular GSH, which has a crucial role in intracellular redox homeostasis and ROS regulation, was evaluated. Au-NPs treatment substantially increased the loss of intracellular GSH level compared with that of the control and He-CAP groups. This increase was also significantly enhanced in the combined groups, Fig. 3d. This finding indicates that the Au-NPs initially decrease intracellular GSH, which facilitated the formation of intracellular ROS in the presence of He-CAP.

Furthermore, it has been well established that enhanced ROS leads to DNA damage in cancer cells. The assessment of the extent of H2AX phosphorylation, as indicated by western blotting, showed that DNA damage was substantially increased in the cells administered combined treatment compared with the cells treated with He-CAP alone (Fig. 3e). Finally, to confirm that the modulation of ROS is involved in the enhancement mechanism of the Au-NPs, cells were pre-treated with *N*-acetyl-l-cysteine (NAC) (5 mM) for 60 min and then irradiated with He-CAP in the presence or absence of Au-NPs. NAC pre-treatment significantly attenuated the enhanced cell death that had been induced by the combined treatment (Fig. 3f, g), which suggests that ROS has a major role in the execution of apoptotic cell death.

### MMP depolarization is enhanced by Au-NPs

**Fig. 4:**
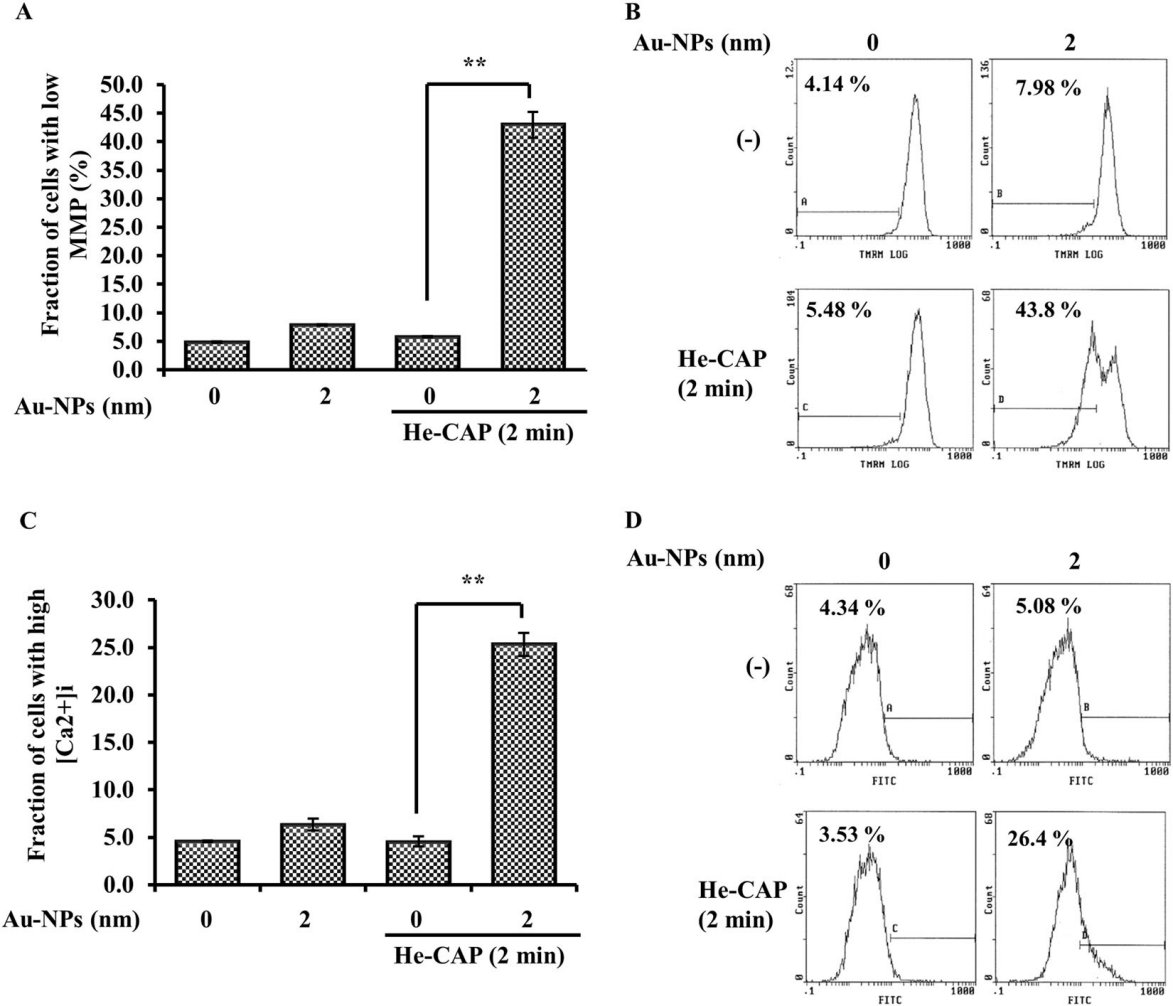
Effects of the Au-NPs on the He-CAP induced intracellular apoptotic pathway. Cells were treated with or without Au-NPs and exposed to He-CAP for 2 min, 18 h after following different treatments, the percentage of MMP loss in these cells, using TMRM staining and intracellular Ca^2+^ level using fluo-3/AM were measured by flow cytometry. **a** Loss of MMP. Results are represented as means ± S.E.M. of three independent experiments, **P < 0.001 compared with the He-CAP alone group evaluated by Student’s *t* test (S.E.M. is indicated by bars). **b** Representative flow cytometry histogram of M.M.P. loss. **c** Intracellular calcium concentration. Results are represented as means ± S.E.M. of three independent experiments, **P < 0.001 compared with the He-CAP alone group evaluated by Student’s *t* test (S.E.M. is indicated by bars). **d** Representative flow cytometry histogram of intracellular calcium level changes.

The fraction of cells in the combined group showed increased loss of MMP 18 h after treatment compared with the MMP status after cell treatment with Au-NPs or He-CAP alone (Fig. 4a, b). We then assessed the combined treatment-induced changes in intracellular calcium (Ca^2+^) homeostasis. Au-NPs treatment alone led to a slight increase in intracellular Ca^2+^ level compared with that of the control, and the He-CAP treatment alone had no effect. However, after the combined treatment, the intracellular Ca^2+^ level increased ~20-fold that of the basal level (5.0 ± 2.0% to 25.0 ± 2.0%), in contrast to the effect of either treatment alone (Fig. 4c, d).

### Au-NPs with He-CAP initiate Fas externalization and caspase-8 activation

**Fig. 5:**
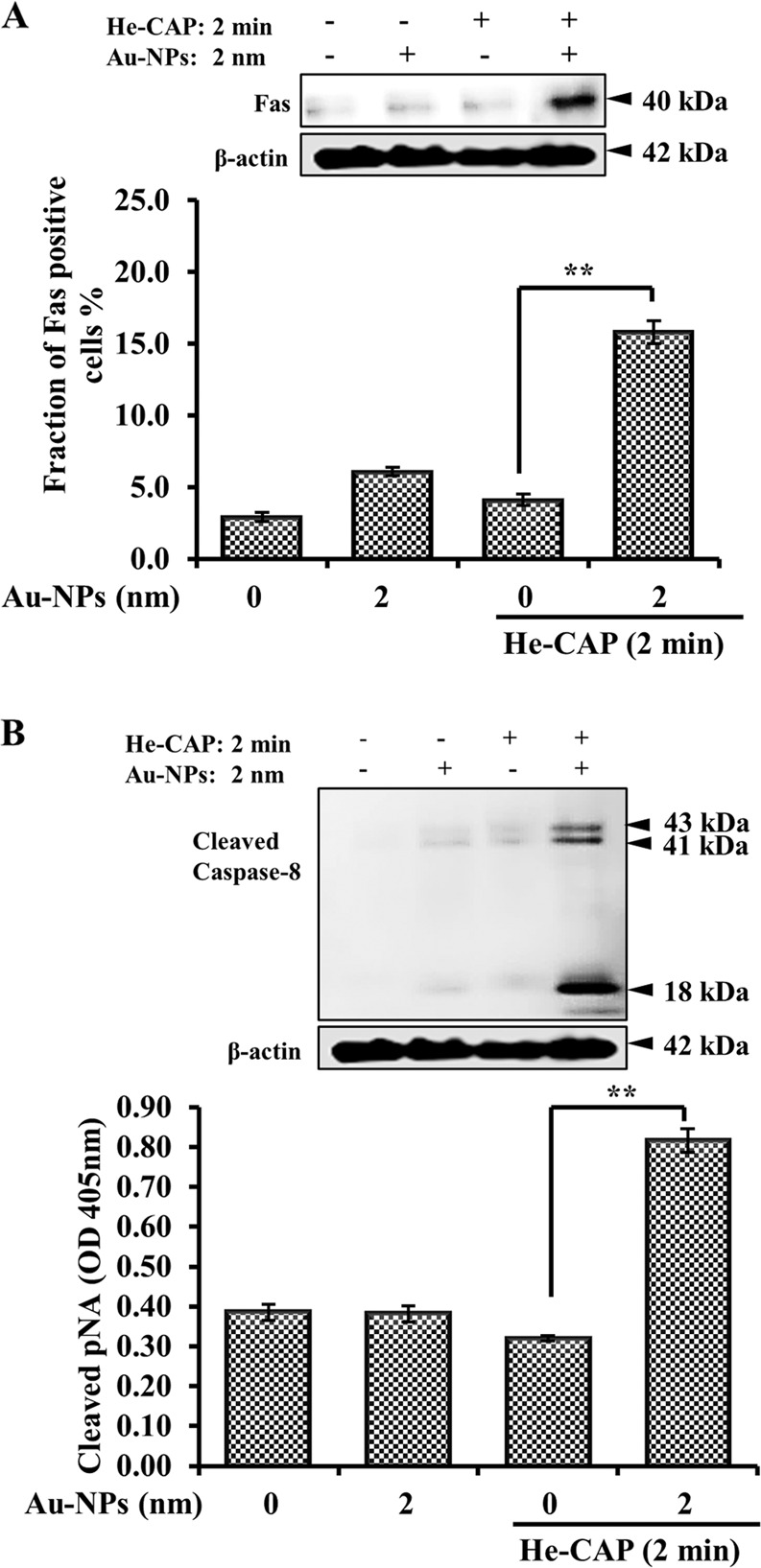
Effects of Au-NPs on the He-CAP-induced extracellular apoptotic pathway. **a** Detection of Fas externalization by western blot analysis and flow cytometry using an anti-Fas FITC-conjugated antibody, 18 h after either treatment. Results are represented as means ± S.E.M. of three independent experiments, **P < 0.001 compared with the He-CAP alone group evaluated by Student’s *t* test (S.E.M. is indicated by bars). **b** Caspase-8 activation and expression in the U937 cells were induced by He-CAP alone and in combined treated cells, measured by a western blotting and a FLICE/caspase-8 colorometric protease kit. Results are represented as means ± S.E.M. of three independent experiments, **P < 0.001 compared with the He-CAP alone group evaluated by Student’s *t* test (S.E.M. is indicated by bars). For western blot β-actin was used to normalize the expression level in each sample. Same β-actin blot was used as the loading control for F.A.S. and cleaved caspase-8 expression. Cropped blots are shown, full-length blots are presented in Supplementary Fig. 3.

Fas can upregulate owing to the formation of ROS, which ultimately leads to apoptosis^15^. FAS receptor activation is linked with the induction of the extrinsic apoptosis pathway through DISC assembly and downstream activation of caspase-8. Hence, it was of interest to examine whether Au-NPs are capable of enhancing Fas externalization and enhancing the He-CAP induced extrinsic apoptosis pathway in cells exposed to He-CAP with or without Au-NPs. The results showed a significant enhancement in Fas externalization only for the combined treatment, as indicated by both the flow cytometry and western blotting results (Fig. 5a). Activation of caspase-8, which is downstream of FAS, is required to initiate the FAS-mediated apoptosis cascade. Consistent with FAS externalization, caspase-8 activation was increased only after the combined treatment was administered, with no marked change after either treatment was administered alone (Fig. 5b).

### Effects of Au-NPs on the expression of apoptotic-related proteins

**Fig. 6:**
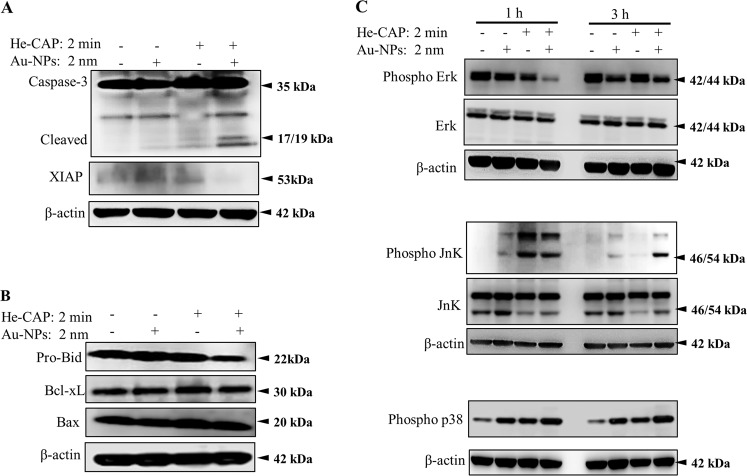
Western blot analysis results showing changes in the expression of apoptosis-related proteins. Cells were treated with or without Au-NPs and then harvested 1, 3, and 24 h after He-CAP treatment. Western blot analysis of **a** caspase-3 and XIAP **b** Bcl-2 family proteins, and **c** MAPK pathway proteins. β-actin was used to normalize the expression level of each sample. Blots were cropped; full-length blots are presented in Supplementary Fig. 4, supplementary Fig. 5 and supplementary Fig. 6.

To gain a mechanistic understanding of the enhancement of apoptosis, we used western blot analysis to evaluate the expression level of several intrinsic apoptosis-associated proteins, including caspase-3 and Bcl-2 family proteins. The combined treatment resulted in a significant increase in cleaved caspase-3 expression compared with that induced by He-CAP or the control alone. For confirmation, we also measured the expression of XIAP and found that it was suppressed in the combined treatment group, suggesting the involvement of caspase-3 in the enhancement of He-CAP induced apoptosis by Au-NPs (Fig. 6a). In addition, the expression of pro-apoptotic pro-Bid (i.e., inactive form) members of the Bcl-2 family was significantly decreased in the combined treatment. The decrease in the pro-Bid form is linked to its activated form truncated-Bid (t-Bid). Unfortunately, we did not observe t-Bid. Similarly, the other Bcl-2 family proteins, such as pro-apoptotic Bax and anti-apoptotic Bcl-xL, remained unchanged (Fig. 6b). These data indicated that Au-NPs could enhance He-CAP induced apoptosis via the intrinsic caspase pathway.

Mitogen-activated protein kinases (MAPKs) also play crucial roles in apoptosis, and oxidative stress activates MAPK family members via protein phosphorylation^16^. The phosphorylation states of JNK (pJNK), ERK1/2 (pERK1/2), and p38 (pp38) were measured by western blotting 1 and 3 h after treatment. pERK1/2 expression was slightly decreased after 1 h of He-CAP treatment alone and was markedly upregulated after 3 h compared to that in the Au-NPs and control groups. Treatment of Au-NPs alone caused no change after 1 h but decreased pERK1/2 expression after 3 h. The combined treatment induced a marked decrease in pERK1/2 expression at 1 h, which was slightly recovered 3 h later but remained lower than that of the cells treated with He-CAP or Au-NPs alone. pJNK expression increased in both the He-CAP alone and combined groups at 1 h, with no significant difference between these two groups. After 3 h, He-CAP induced pJNK expression decreased, but it remained elevated in the combined group. Similarly, p38 expression was also markedly elevated in the combined group at 1 and 3 h compared with that of either treatment alone. In summary, no marked changes were observed in the expression of ERK1/2 and JNK (Fig. 6c).

## Discussion

CAP has the ability to induce ROS and RNS in both air and liquid environment. CAP induced an enormous amount of ROS in the extracellular milieu. However, its limited interaction with or penetrability into cells remains a challenge to CAP applications. To address this problem, a multimodality strategy was recently adopted by combining nanoparticles carrying drugs with CAP. Previously, we showed the synergism in He-CAP induced apoptosis upon exposure to hyperthermia and sulfasalazine^17,18^. Here, we demonstrated the size-dependent effects of 2 nm, 40 nm, and 100 nm Au-NPs. It has been established that the size and shape of Au-NPs are essential factors because the internalization of Au-NPs in a tumor may depend on their size and surface properties.

Here, the initial experiments showed that Au-NPs of size 40 and 100 nm did not enhance He-CAP-induced apoptosis. In contrast, only 2 nm Au-NPs synergistically enhanced He-CAP-induced apoptosis. These findings are different from those of previous studies that indicated a potential synergy between Au-NPs and CAP. CAP treatment with citrate-capped Au-NPs of 20 nm led to the synergistic cell death of U373MG cells by increasing Au-NPs endocytosis^19^. Similarly, CAP with 100 nm Au-NPs induced up-to a 30% overall increase in glioblastoma (U87) cell death, and Au-NPs of 55 nm induced cell death in colorectal cancer cells^10,11^. In addition, melanoma cell death increased about fivefold in the presence of antibody-conjugated 30 nm Au-NPs and the proliferation of glioblastoma multiform, lung adenocarcinoma cells was suppressed when treated with 100 nm PEG-coated Au-NPs^20,21^. Our finding did not wholly contradict these previously published studies as the differences in numerous variables, including cell type, concentration, surface coating, CAP device, and exposure time cannot be neglected. However, it is important to note that the biological action of 2 nm Au-NPs is different than that of larger Au-NPs (40 nm and 100 nm) when administered with He-CAP, as revealed here for the first time.

He-CAP induced a higher level of hydroxyl radical (^•^OH) in the liquid phase, and when Au-NPs were exposed to He-CAP the electron spin resonance spectra for DMPO-OH adducts did not markedly increase (data not shown). This finding indicates that the combined Au-NPs and He-CAP treatment had no effect on ^•^OH formation in the liquid phase. In contrast, cells treated with He-CAP in the presence of Au-NPs showed a marked increase in intracellular ROS formation. In this study, 3 h after the combined treatment was administered, an elevation in intracellular ROS was observed using DCFH-DA. In addition, He-CAP alone increased O_2_^•−^ generation but failed to initiate apoptosis because the effect lasted for a short time. In contrast, the combined treatment led to a more sustained increase in O_2_^•−^ levels, which corresponded with apoptosis induction. Importantly, we also observed intracellular ROS through the use of using HPF dye, which is specific mainly for ^•^OH but can also be used to detect generated peroxynitrite (ONOO^−^). The combined treatment significantly increased the HPF intensity compared with that of either treatment alone (Fig. 3a–c). Although nitric oxide (NO) was not detected directly, excess O_2_^•−^ is known to react with NO and ultimately generate the highly reactive oxidant ONOO^−^^22^. Therefore, we assumed that the combined treatment mediated the cytotoxic effects via NO and subsequent ONOO^−^ generation. These findings confirm the cell-nanoparticle interactions as a sustained elevation of intracellular oxidative stress is linked to cellular processes such as mitochondrial respiratory chain reaction^23^. It is well known that intracellular GSH is one of the major scavenger of ROS and serves as a marker of oxidative stress^24^.

Here, it was found that Au-NPs alone decrease the intracellular GSH, thus making cells more susceptible to ROS generation. Consistently, the combined treatment significantly decreases intracellular GSH synthesis and lead to increase in ROS generation. This outcome is the result of several factors. First, GSH (thiol bonds) binds preferentially and firmly to the gold surface. If the gold size is smaller than that of the protein, Au-NPs can bind to thiol groups without steric hindrance, and the chemical reaction rate coefficients are high. Second, Au-NPs have size-dependent effects. These effects are caused by Au-NPs, with primarily metal characteristics, transitioning into particle insulator characteristics. Thus, a catalytic chemical reaction is induced by the electron transfer between the reagent and substrate. This characteristic is maximized with ~100 Au atoms on an NP, giving it a size of 2 nm^25,26^. Au-NPs of smaller sizes has been reported to enter into the nucleus of cancer cells^27^. However, in this study, Au-NPs alone did not increase H2AX expression, which suggests that the biological sensitization was mainly owing to the localization of Au-NPs within the cytoplasm, thus limiting any DNA-damaging capabilities and confirming the notion that DNA damage is secondary to intracellular ROS formation (Fig. 3e). The most likely reason for the synergistic enhancement of 2 nm Au-NPs, whereas those of 40 nm and 100 nm were infective, relates to the 2 nm Au-NPs being endocytosed more rapidly and at a higher rate than the large Au-NPs and localizing into cytoplasmic vesicles with less exposure time, increasing their opportunity to decrease intracellular GSH, and thus trapping more CAP-induced reactive species through their extended half-life^28,29^. CAP has been reported to accelerate Au-NPs cellular uptake^30^. It is not surprising that physiochemical differences in nanoparticles, especially size, can affect their uptake efficiency, kinetics, and intracellular localization^31^. Several studies confirmed the higher uptake and internalization efficiency of smaller Au-NPs^32,33^. Small size nanoparticles provide advantages for the passive targeting of cancer cells because of the enhanced EPR effect, and smaller size of NPs is associated with deeper intracellular penetration and the induction of more significant cytotoxic effects^34,35^. Au-NPs size-dependent cytotoxicity has been reported previously, and similar Au-NPs with only a difference in size showed varied cytotoxic profiles. Au-NPs of 1.4 nm were more toxic than those of 15 nm^36^.

Elevated intracellular ROS formation can damage to intracellular components and promote apoptosis through intrinsic and extrinsic signaling pathways^37,38^. The combined treatment induced a large amount of intracellular ROS formation, which activated the intrinsic pathway of apoptosis. In addition, ROS also induced apoptosis by Fas upregulation^15^. FAS activity was slightly increased upon Au-NPs treatment alone compared with that induced by the control or the He-CAP alone treatment. However, the Au-NPs treatment alone failed to induce downstream of FAS, caspase-8 activation. In contrast, the combined treatment significantly increased the activation of both FAS and caspase-8. Therefore, it was assumed that, although the Au-NPs initially induced FAS activation, it was maintained at a minimum threshold level. In contrast, the combined treatment caused further increases in Fas activity, owing to the interaction of the increased number of intracellular ROS, and ultimately induced caspase-8 activation. The FAS-mediated cell death pathway induces direct induction of the caspase cascade or activation of downstream caspase-8. In turn, caspase-8 executes apoptosis through Bid activation^39^.

The data from this study showed a positive correlation between intracellular ROS modulation through a combined treatment and apoptosis induction. Oxidative and cellular stresses can activate the MAPKs pathway, which also has a principal role in the induction of apoptosis^16^. In this study, pJNK was markedly increased following administration of the combined treatment compared with either treatment alone at 3 h, although He-CAP alone increased pJNK at 1 h, it was not substantially changed compared with the pJNK increases induced by the combined treatment. We speculate that this enhanced apoptosis is because of JNK activity as it subsided after the He-CAP alone treatment but remained elevated in the combined treatment after 3 h. Consistently, the increase in pERK after both 1 and 3 h of treatment suggested that ERK is activated in response to the Au-NPs and He-CAP treatment. However, the marked decrease in pERK at 1 h and 3 h after exposure to the combined treatment, compared with that of either treatment alone indicated decreased survival ability, because of apoptosis. Finally, we confirmed that the modulation of ROS was the critical factor in the synergistic enhancement induced by the combined treatment. NAC was administered and attenuated the enhanced cell death significantly. This finding confirms that ROS has significant role in the execution of enhanced apoptotic cell death.

**Fig. 7:**
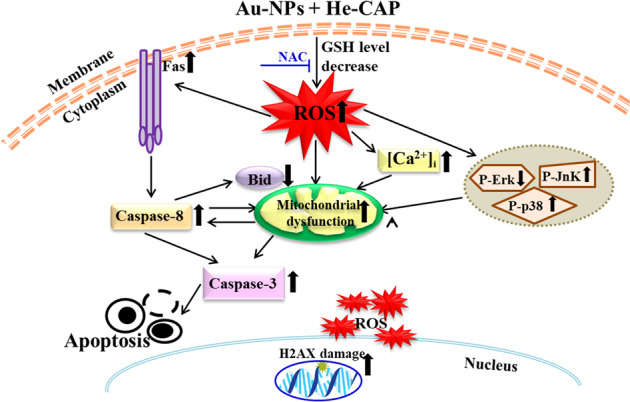
Small Au-NPs decrease intracellular glutathione levels and lead to excessive intracellular ROS generation. Au-NPs/He-CAP co-treatment induces apoptosis via both intrinsic and extrinsic signaling pathways.

In summary, only small Au-NPs can enhance He-CAP induced apoptosis owing to the decrease of intracellular GSH levels that leads to excessive generation of intracellular ROS. ROS-mediated damage to intracellular components and cell surface death receptor activation initiate apoptotic cell death either by intrinsic or extrinsic apoptotic pathways (Fig. 7). Here, it is important to note that the data were obtained using a single cell line, and further evaluation using solid tumor cell lines would be needed in the future. However, the findings presented in this study highlight the importance of determining the Au-NPs size-dependent effects with CAP on various cancer cells. The complex phenomena and contradictory reports on Au-NPs size make it essential to develop an optimized design of Au-NPs for future CAP therapy. In addition, it is crucial to carefully select appropriate Au-NPs size to acquire better therapeutic outcomes with CAP.

## Supplementary information

Figure S1

Figure S2

Figure S3

Figure S4

Figure S5

Figure S6

Supplementary Figure Legends

## Data Availability

Data are available from the authors upon request.
