# Supplementary figures and images for: Small size gold nanoparticles enhance apoptosis-induced by cold atmospheric plasma via depletion of intracellular GSH and modification of oxidative stress
Source: Cell Death Discov. 2020 Sep 10;6:83. doi: 10.1038/s41420-020-00314-x (PMC7483448; doi:10.1038/s41420-020-00314-x)

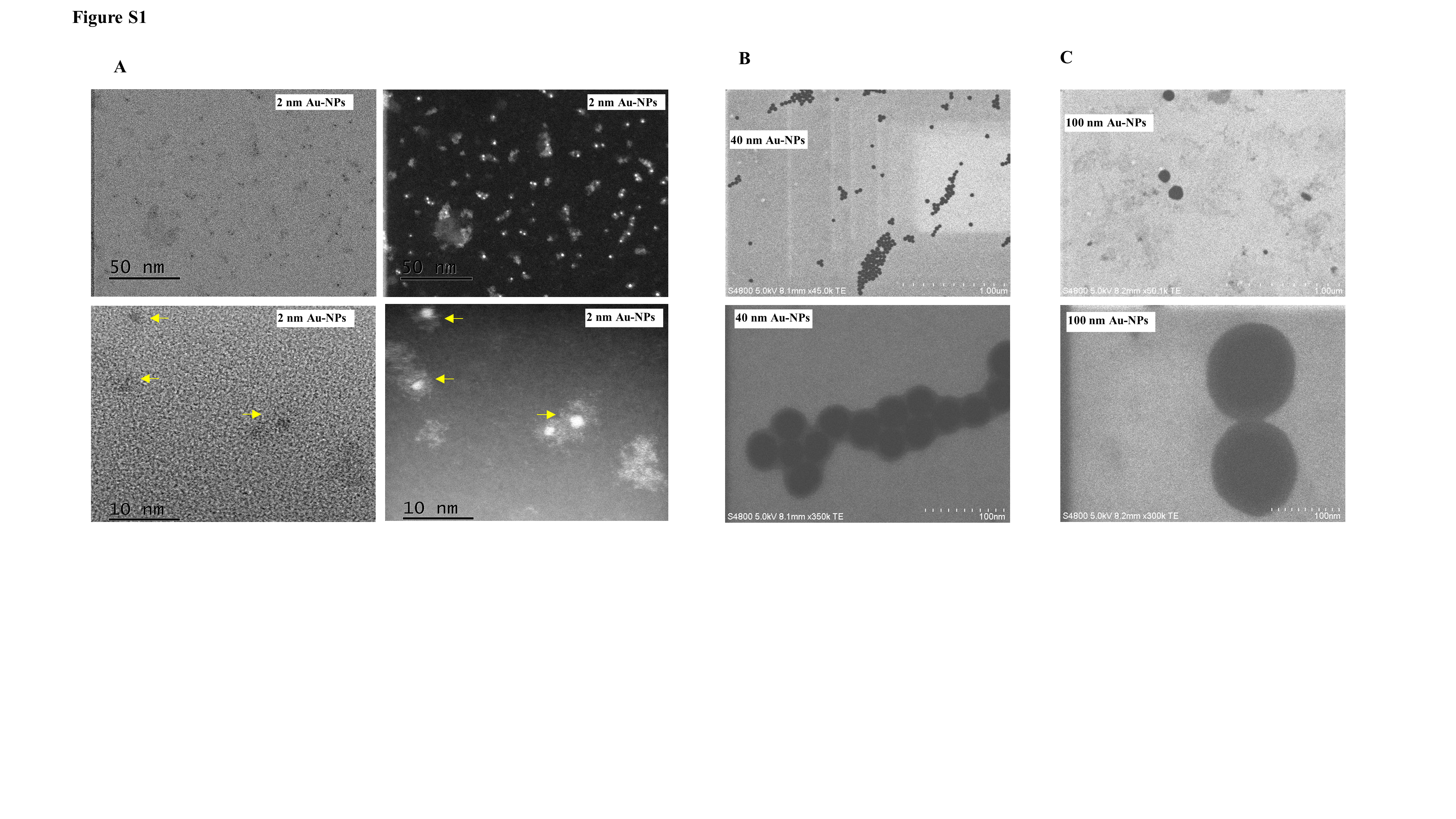

Supplement: Supplementary file 1 — Figure S1 [file 41420_2020_314_MOESM1_ESM.tif]

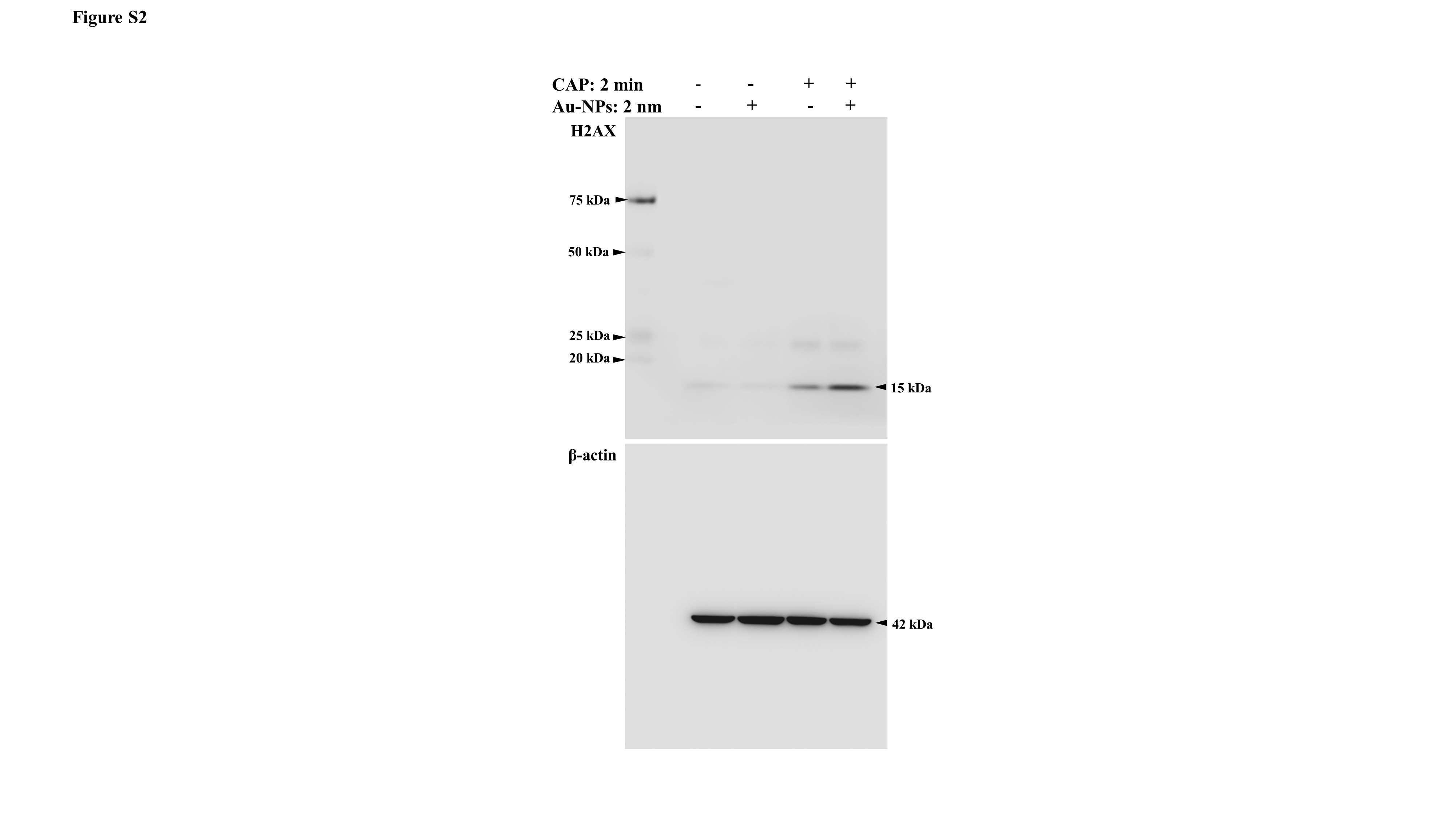

Supplement: Supplementary file 2 — Figure S2 [file 41420_2020_314_MOESM2_ESM.tif]

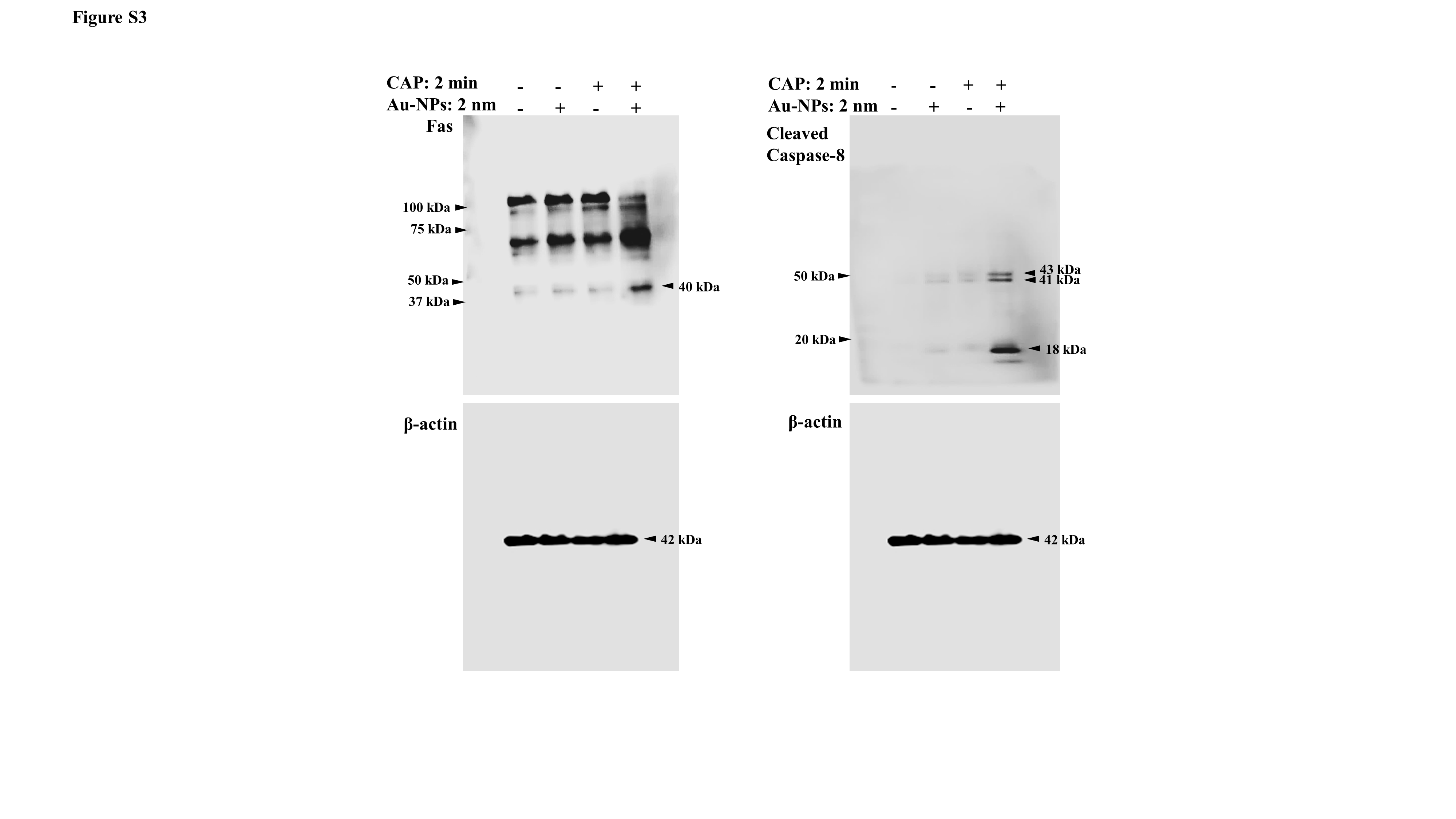

Supplement: Supplementary file 3 — Figure S3 [file 41420_2020_314_MOESM3_ESM.tif]

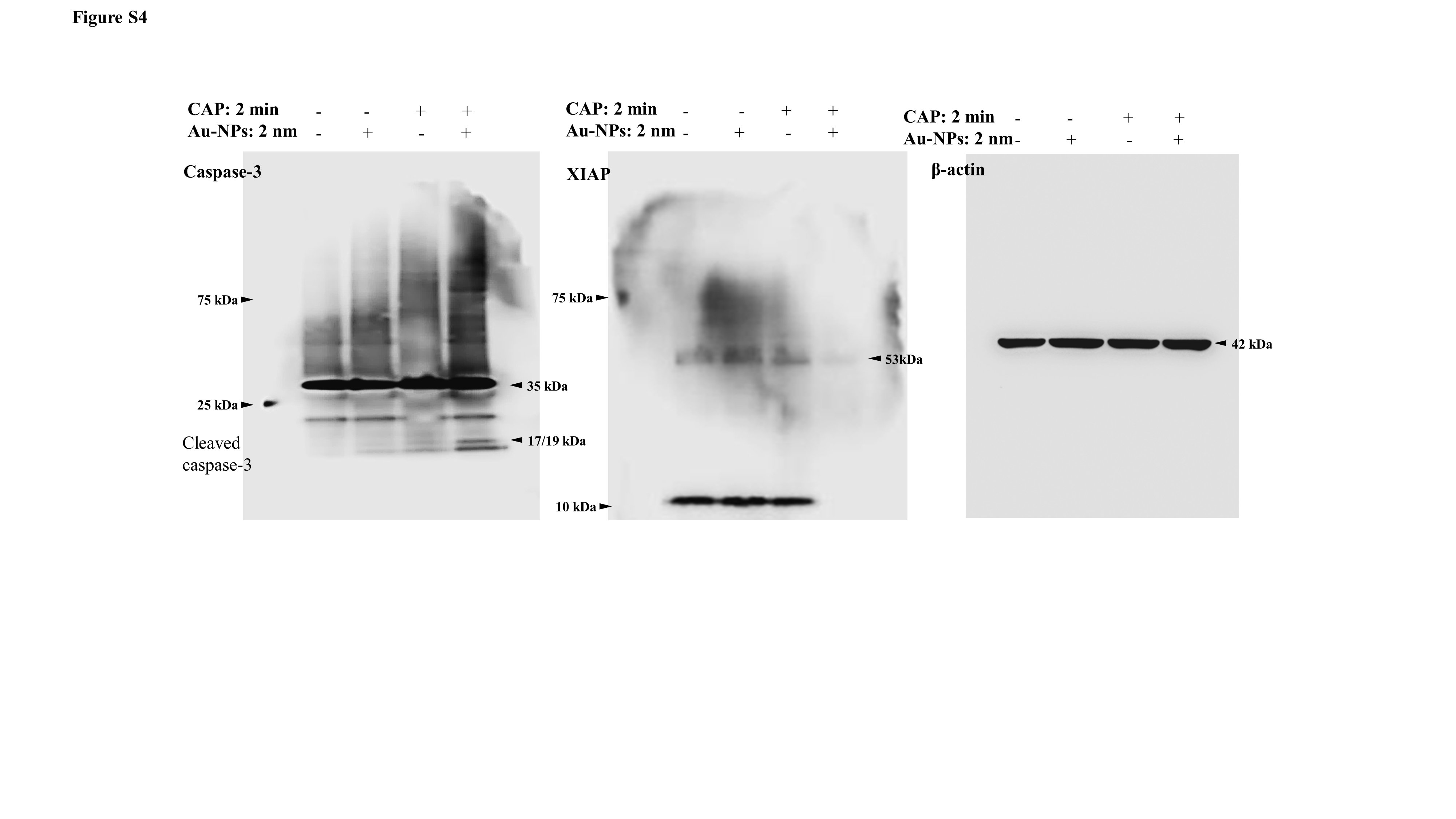

Supplement: Supplementary file 4 — Figure S4 [file 41420_2020_314_MOESM4_ESM.tif]

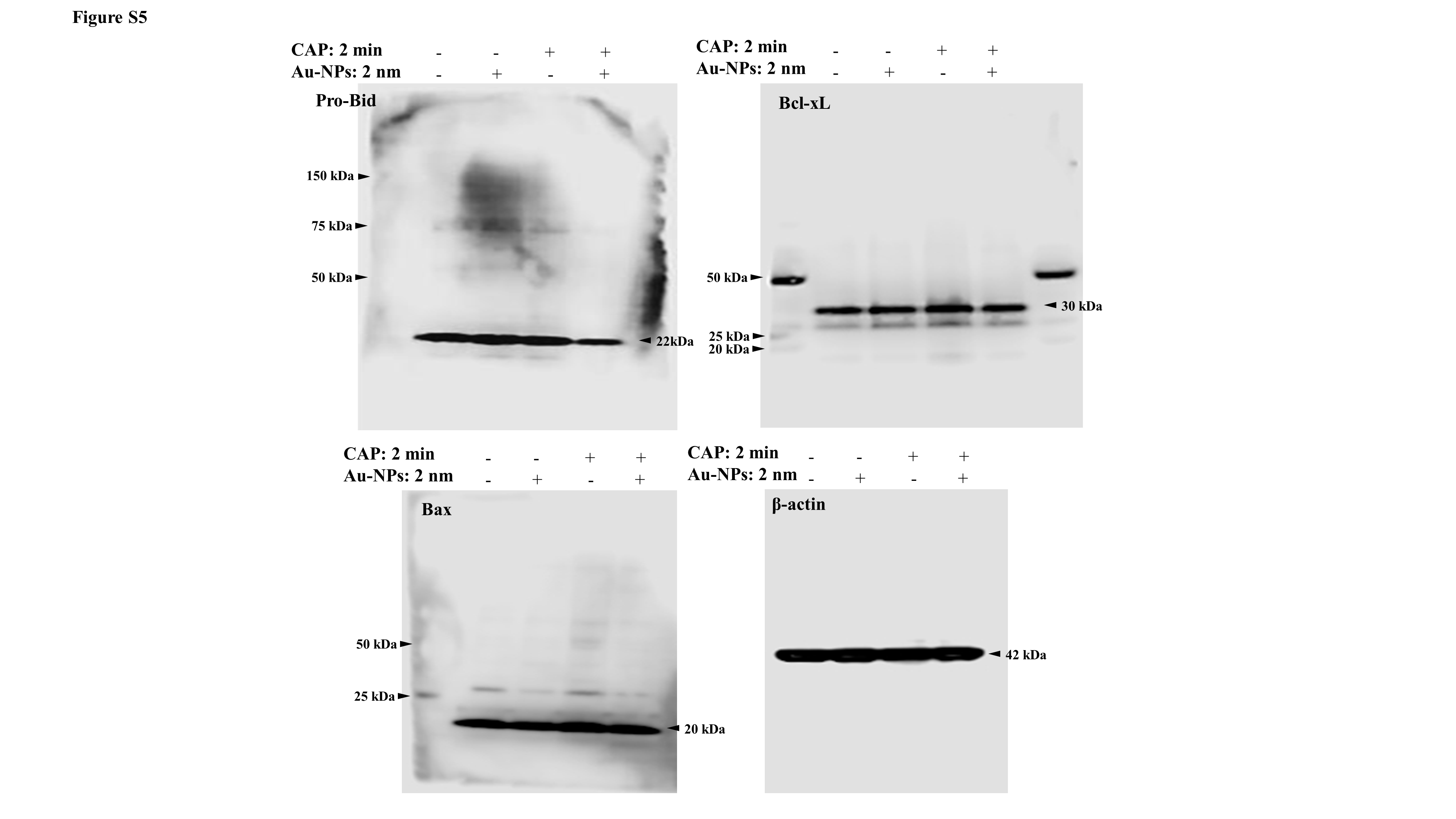

Supplement: Supplementary file 5 — Figure S5 [file 41420_2020_314_MOESM5_ESM.tif]

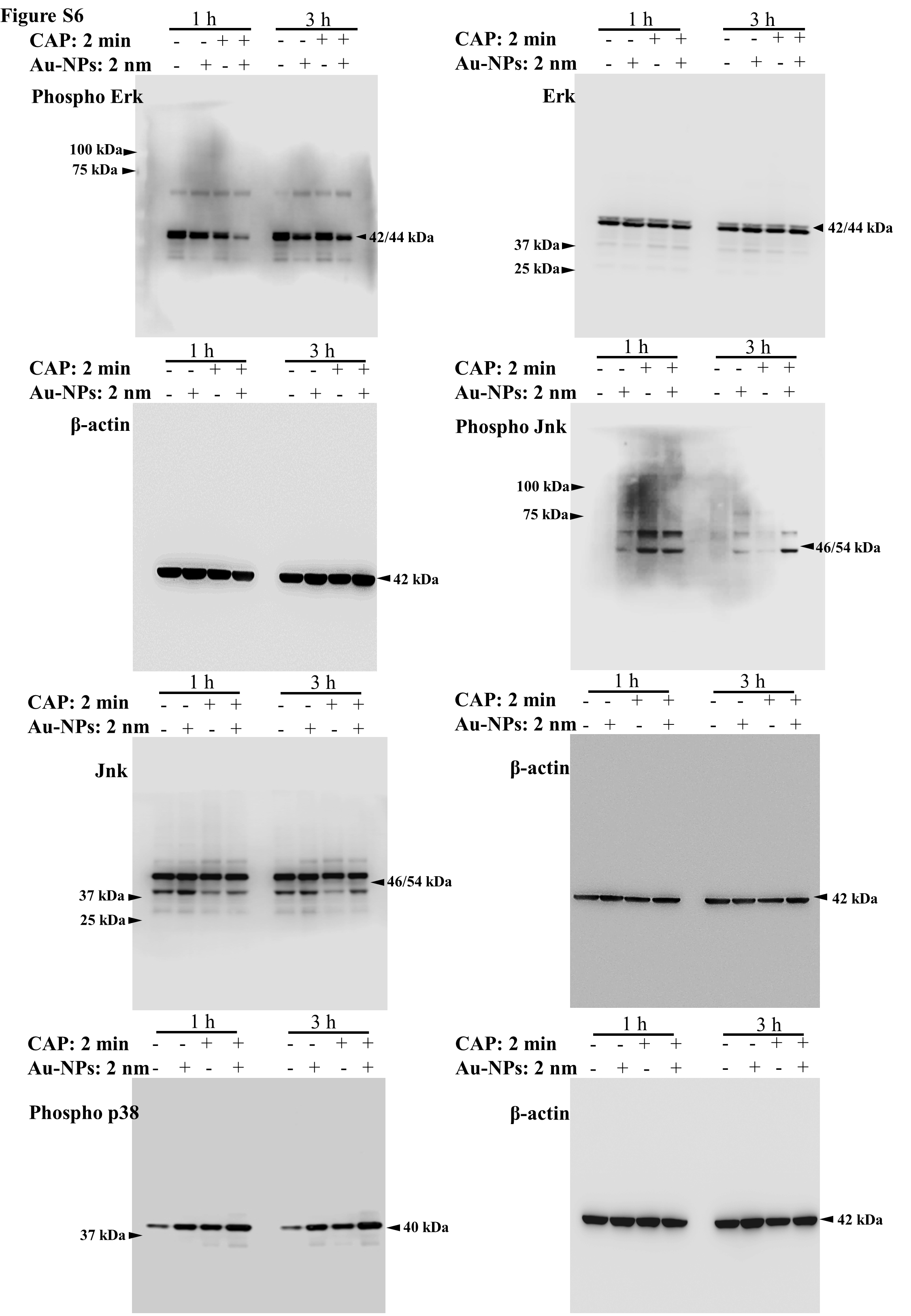

Supplement: Supplementary file 6 — Figure S6 [file 41420_2020_314_MOESM6_ESM.tif]
