# Supplementary material for: Small size gold nanoparticles enhance apoptosis-induced by cold atmospheric plasma via depletion of intracellular GSH and modification of oxidative stress
Source: Cell Death Discov. 2020 Sep 10;6:83. doi: 10.1038/s41420-020-00314-x (PMC7483448; doi:10.1038/s41420-020-00314-x)
Supplement: Supplementary file 7 — Supplementary Figure Legends [file 41420_2020_314_MOESM7_ESM.docx]

**Fig. S1.** Transmission electron microscopy images of gold nanoparticles with sizes **(A)** 2 nm, Scale bars, upper panel 50 nm and lower panel 10 nm. Arrow heads shows Au-NPs of size 2 nm **(B)** 40 nm, Scale bars, upper panel 1.0 μm and lower panel 100 nm **(C)** 100 nm. Scale bars, upper panel 1.0 μm and lower panel 100 nm as shown.

**Fig. S2.** Full-lengths blots of Figure 3E.

**Fig. S3.** Full-lengths blots of Figure 5A & B.

**Fig. S4.** Full-lengths blots of Figure 6A.

**Fig. S5.** Full-lengths blots of Figure 6B.

**Fig. S6.** Full-lengths blots of Figure 6C.
